# Proliferation of Interstitial Cells in the Cyclophosphamide-Induced Cystitis and the Preventive Effect of Imatinib

**DOI:** 10.1155/2017/3457093

**Published:** 2017-06-18

**Authors:** Maria Sancho, Domingo Triguero, Aranzazu Lafuente-Sanchis, Angeles Garcia-Pascual

**Affiliations:** ^1^Department of Physiology, School of Veterinary Medicine, Complutense University, Madrid, Spain; ^2^Department of Physiology & Pharmacology, University of Western Ontario, London, ON, Canada

## Abstract

Cyclophosphamide- (CYP-) induced cystitis in the rat is a well-known model of bladder inflammation that leads to an overactive bladder, a process that appears to involve enhanced nitric oxide (NO) production. We investigated the changes in the number and distribution of interstitial cells (ICs) and in the expression of endothelial NO synthase (eNOS) in the bladder and urethra of rats subjected to either intermediate or chronic CYP treatment. Pronounced hyperplasia and hypertrophy of ICs were evident within the lamina propria and in the muscle layer. IC immunolabeling with CD34, PDGFR*α*, and vimentin was enhanced, as reflected by higher colocalization indexes of the distinct pairs of markers. Moreover, de novo expression of eNOS was evident in vimentin and CD34 positive ICs. Pretreatment with the receptor tyrosine kinase inhibitor Imatinib prevented eNOS expression and ICs proliferation, as well as the increased voiding frequency and urinary tract weight provoked by CYP. As similar results were obtained in the urethra, urethritis may contribute to the uropathology of CYP-induced cystitis.

## 1. Introduction

Hemorrhagic cystitis is a well-known adverse effect of cyclophosphamide (CYP), a cytotoxic alkylating agent used to treat various malignant tumors and autoimmune diseases. In experimental animals, CYP-induced cystitis is a common model of bladder inflammation in which symptoms of an overactive bladder (OAB) develop [1, 2]. CYP-induced cystitis is characterized by interstitial cystitis, accompanied by an increase in the micturition frequency and in the number of low volume voids [3, 4]. An increase of nitric oxide (NO) production is implicated in the pathogenicity of CYP-induced cystitis, and this is generally assumed to result from the upregulated expression of the inducible isoform of NO synthase (iNOS) [1]. However, we also reported a progressive upregulation of endothelial NOS (eNOS) in CYP-treated rats that could alternatively explain the increased NO production during cystitis [4].

Interstitial cells (ICs), a phenotype originally compared to the gastrointestinal interstitial cells of Cajal (ICCs) [5], are found throughout in the lower urinary tract. In the urethra, ICs act as pacemakers of the basal tone that maintains continence [6] and as mediators of the relaxant nitrergic neurotransmission that triggers micturition [7, 8]. Likewise, they seem to be involved in cholinergic neurotransmission of the bladder [9], detrusor excitability [10], and sensory function of the bladder lamina propria [11].

Several reports indicate a direct relationship between urinary bladder dysfunction and ICs. An increase in ICs was reported in a model of bladder obstruction [12], and an altered distribution and/or phenotypic disturbances of ICs may contribute to the pathophysiology of OAB [13]. However, the effect of CYP-induced cystitis on bladder and urethral ICs has yet to be elucidated. Imatinib, a receptor tyrosine kinase inhibitor currently used to treat lymphomas and c-kit^+^ solid tumors of the gastrointestinal tract, is known to inhibit the c-kit and PDGF receptors [14]. In the bladder, Imatinib reduces spontaneous and induced bladder activity, an effect that appears to be more prominent in OAB syndrome [15–18]. Furthermore, the effects of Imatinib on the bladder are thought to be mediated through ICs, thereby blocking the cholinergic transmission of signals to the bladder [19].

Accordingly, here we have studied the changes in the number and distribution of ICs in the bladder and urethra of rats subjected to intermediate (48 h) or chronic (10 days) CYP treatment, quantifying the ICs immunolabeled by pairs composed of the following markers: vimentin, CD34, and platelet derived growth factor receptor alpha (PDGFR*α*). Moreover, the expression of eNOS and its relationship to these cells were also assessed. Finally, we tested the effect of the pretreatment with Imatinib on micturition frequency and urinary tract wet weight and on the expression of the different pair of ICs markers and eNOS. Our results highlight the proliferation of ICs in association with CYP-induced cystitis and emphasize the potential of Imatinib as a specific inhibitor of IC activity and, hence, its possible application in experimental and clinical medicine.

## 2. Material and Methods

### 2.1. CYP and Imatinib Treatment

Hemorrhagic cystitis was induced in 48 adult female Wistar rats (200–250 g) by administering cyclophosphamide monohydrate (CYP; Sigma Chemie GMbH, Steinheim, Germany) dissolved in NaCl 0.9%. The rats were housed individually (12 h light/dark cycle) with ad libitum access to food and water, and CYP was administered according to the following regimes: (i) intermediate treatment: 150 mg kg^−1^ i.p. 48 h before sacrificing or (ii) chronic treatment: 50 mg kg^−1^ i.p. every 3 days for 10 days. Control animals (*n* = 12) received a corresponding volume of saline (0.9%). A separate group (*n* = 24) was given Imatinib orally five days before and during both CYP treatments (10 mg kg^−1^, Imatinib mesylate, LC Laboratories, USA). All procedures were approved by the Ethical Committee at the Complutense University and they were performed in accordance with European Council Directive 2010/63/EU.

### 2.2. Voiding Frequency Test

Micturition frequency was analyzed in all the rats, as described previously [4]. Drinking water was removed one hour before testing and the rats were left in cages lined with filter paper for 30 min. A UV light source was used to visualize and trace the urine spots on the filter paper, registering both the total number of urine spots and those of small diameter (<0.5 cm), expressed as the number of voids per hour.

### 2.3. Immunofluorescence on Cryostat Sections

Animals were anesthetized (40 mg kg^−1^ ketamine + 5 mg kg^−1^ xylazine, i.p.) and then subjected to cardiac perfusion with heparinized 0.1 M phosphate buffer (PB), followed by 4% paraformaldehyde in PB for 30 min. The lower urinary tract was removed and weighed (wet), and tissue samples (5 × 5 mm) were obtained from the middetrusor or the proximal urethra and fixed in ice-cold 4% paraformaldehyde in 0.1 M PB (pH 7.0). As described previously [8], the tissue was cryoprotected in increasing concentrations of sucrose (10–30%) and then snap-frozen in liquid nitrogen-cooled isopentane. Cryostat sections transverse to the mucosal surface (7 *μ*m: CM1850 UV, Leica Microsystems, Barcelona, Spain) were recovered on poly-L-lysine-coated slides and immunofluorescent staining was performed using the following primary antibodies: mouse monoclonal anti-vimentin (clone V9, 1 : 200; Chemicon International); rabbit polyclonal anti-c-kit (C-19); goat polyclonal anti-CD34; and rabbit polyclonal anti-PDGFR*α* (all at 1 : 100; Santa Cruz Biotechnology, Santa Cruz, CA); rabbit polyclonal anti-eNOS (1 : 100; Cayman Chemical, USA). These antibodies were detected with the following secondary antibodies: Alexa Fluor 488 donkey anti-rabbit, Alexa 488 donkey anti-goat, Alexa 594 donkey anti-rabbit, and Alexa 594 donkey anti-mouse (all at 1 : 200; Molecular Probes, Eugene, OR). The nuclei were counterstained with 4′,6-diamino-2-phenylindole (DAPI, 10.9 mM; Sigma-Aldrich) and the sections were mounted with Prolong Gold® antifade reagent (Molecular Probes, Eugene, OR, USA). In all cases, negative controls were run in parallel in which the primary antibody was omitted.

Sections were visualized on an Axioplan 2 fluorescence microscope (Carl Zeiss Microimaging, Göttingen, Germany) and photographed with a Spot-2 digital camera (Diagnostic Instruments Sterling Heights, MI, USA). The images were stored digitally as 12-bit images using MetaMorph 6.1 software (MDS Analytical Technologies, Toronto, ON, Canada) and the intensity of the immunofluorescence was quantified as described previously [8]. A threshold was established to subtract the background immunofluorescence and the proportion of the selected area where the staining exceeding the threshold value was analyzed separately in the lamina propria and muscle layer. To avoid interference from edema, measurements were normalized according to the number of cells in the area of interest, dividing by the proportion of the area stained with DAPI. The degree of marker colocalization in dual immunofluorescence was determined with the Colocalization Colormap plugin in the Fiji software package, which calculates the correlation index (ranged from 0 to 1) as an indication of the positively correlated pixels within an image [20].

Individual cell sizes were measured in random samples in each region and experimental condition (3-4 cells/slide). At the highest magnification (40x), cell contour was automatically set and surface quantified using ImageJ software.

### 2.4. Statistical Analysis

The data are expressed as the means (±SEM) and they were compared by one-way ANOVA followed by a Dunnett's multiple comparison test. *P* values ≤ 0.05 were considered statistically significant.

## 3. Results

### 3.1. Changes in the Density and Distribution of ICs Induced by CYP

**Figure 1:**
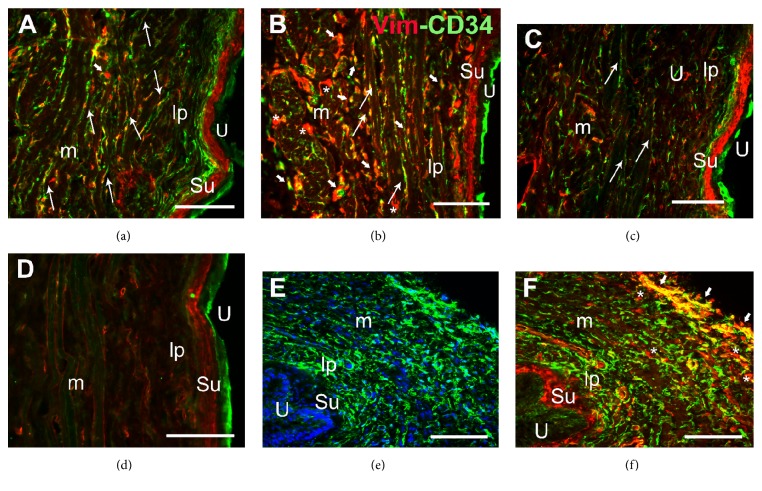
Increased vimentin/CD34 colabeling induced by CYP in the bladder and urethra and the preventive effect of Imatinib. Bladder sections of control (a), CYP-chronic (b), Imatinib-control (c), and Imatinib-chronic (d) treated animals. Labeling for CD34 (green) and vimentin (Vim, red) increased in intensity and extension in the CYP treatments and Imatinib prevented these effects. (e-f) Labeling for CD34 (e) and the merged image Vim-CD34 (f) of a urethral section from a CYP-chronic treated rat. The nuclei were counterstained with DAPI (blue) in (e). Long arrows indicate double-labeled bipolar cells with long prolongations situated parallel to smooth muscle fibers. Short arrows show large double-labeled multipolar big cells in between muscle fibers and in the serosal coat. Asterisks indicate large cells that were only vimentin-immunoreactive. U, urothelium; Su, suburothelium; lp, lamina propria; m, muscle layer. Scale bars = 40 *μ*m.

**Figure 2:**
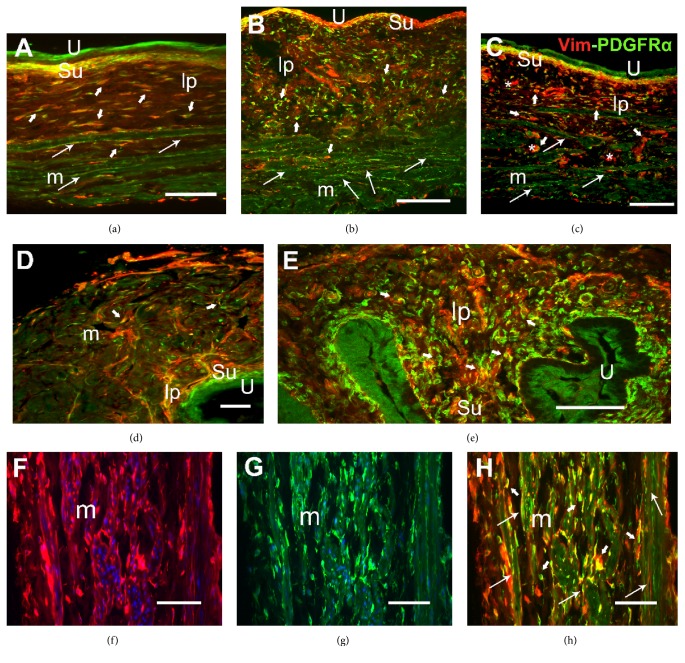
Increased vimentin/PDGFR*α* colabeling induced by CYP in the bladder and urethra and the preventive effect of Imatinib. (a–c) Bladder sections of control (a), CYP-intermediate (b), and CYP-chronic (c) treated animals. (d-e) Urethral sections of control (d) and CYP-intermediate (e) treated animals. Vimentin (Vim, red) and PDGFR*α* labeling (green) increased in intensity and extension in the CYP treatments, effects that were prevented by Imatinib. (f–h) vimentin-ir (f) and PDGFR*α*-ir (g) and the merged images (h) in sections of the bladder from a CYP-chronic treated animal. The nuclei were counterstained with DAPI (blue) in (f) and (g). Long arrows indicate double-labeled bipolar cells with long prolongations. Short arrows show double-labeled multipolar cells present in between muscle fibers and in the lamina propria. Asterisks indicate large cells that were only vimentin-ir. U, urothelium; Su, suburothelium; lp, lamina propria; m, muscle layer. Scale bars = 40 *μ*m except in (b), (c), and (d) (80 *μ*m).

**Figure 3:**
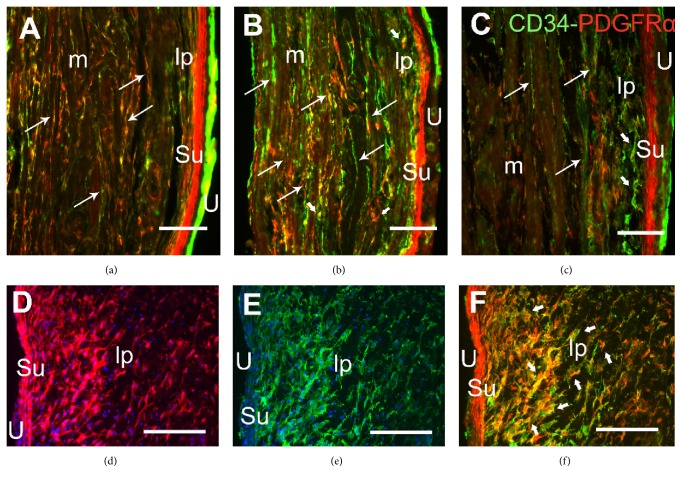
Increased CD34/PDGFR*α* colabeling induced by CYP in the bladder and urethra and the preventive effect of Imatinib. (a–c) Bladder sections of control (a), CYP-intermediate (b), and Imatinib-intermediate (d) treated animals. Labeling for CD34 (green) and PDGFR*α* (red) increased in intensity and extension in the CYP treatments, effects that were prevented by Imatinib. Labeling for PDGFR*α* (d) and CD34 (e) and the merged image (f) in the bladder lamina propria after CYP-intermediate treatment. Nuclei were counterstained with DAPI (blue) in (d) and (e). Long arrows indicate double-labeled bipolar TCs with long prolongations. Short arrows show large double-labeled multipolar cells present in between muscle fibers and in the lamina propria. U, urothelium; Su, suburothelium; lp, lamina propria; m, muscle layer. Scale bars = 40 *μ*m.

**Figure 4:**
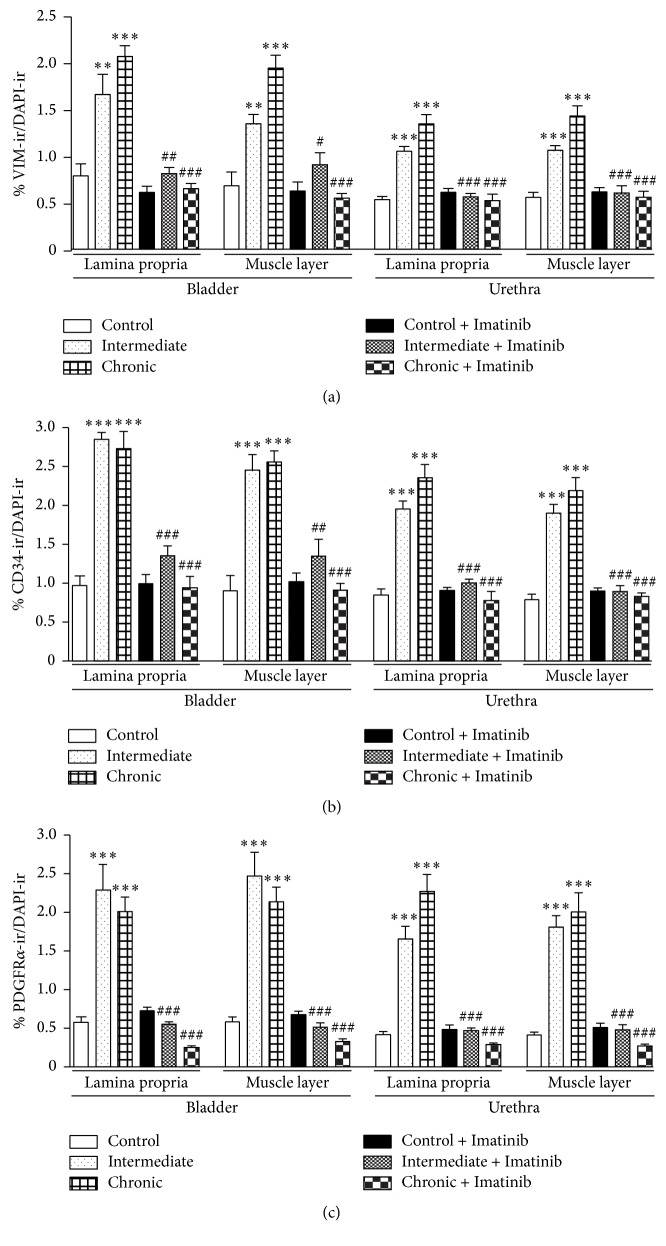
Quantification of vimentin (a), CD34 (b), and PDGFR*α* (c) immunoreactivity in the bladder and urethra of rats given CYP and Imatinib. The staining area above the intensity threshold was measured in hand drawn fields of bladder and urethral sections. Measurements were made independently in the lamina propria and the muscle layer of both organs. Values represent the mean ± SEM (*n* = 6-7 different fields from at least 4 different animals): ^*∗∗*^*P* < 0.01 and ^*∗∗∗*^*P* < 0.001 compared to controls; ^#^*P* < 0.05, ^##^*P* < 0.01, and ^###^*P* < 0.001 compared to the corresponding treatment without Imatinib (one-way ANOVA followed by Dunnett's multiple comparison test).

In both the bladder and urethra, immunoreactivity (ir) to the three markers analyzed was evident in cells that were irregularly distributed throughout the interstitium of the lamina propria, the muscle layer, and the serosal coat. These cells were showed by dual immunolabeling with the following pairs of markers: vimentin/CD34 (Figure 1), vimentin/PDGFR*α* (Figure 2), and CD34/PDGFR*α* (Figure 3). Treatment with CYP provoked changes in the morphology and distribution of these ICs relative to the control animals, both following intermediate (150 mg kg^−1^ for 48 h) or chronic (10 days, 50 mg kg^−1^ every three days) treatment (Figures 1–4).

Among the ICs immunolabeled for CD34, vimentin, and PDGFR*α* in both the bladder and urethra of control animals, there were slender bipolar cells (long arrows in Figures 1–3) that lay along the smooth muscle cells (SMCs), as well as multipolar ICs in the lamina propria and in between the muscle fibers (short arrows in Figures 1–3). In addition, vimentin and PDGFR*α* strongly labeled a dense coat of subepithelial bipolar cells located beneath and parallel to the urothelium in the bladder (Figures 1(a) and 2(a)), while in the urethra these cells were only PDGFR*α*-immunoreactive (Figure 2(d)). Superficial urothelial cells were double-labeled for CD34/PDGFR*α* (Figures 1(a), 2(a), and 3(a)). In our hands and using an antibody distinct to that which previously failed to detect c-kit in the rat urethra (C-19, Santa Cruz Biotechnology) [8], we failed again in detecting specific c-kit staining in ICs both in bladder and in urethral paraformaldehyde fixed sections. c-kit expression was detected in the nucleus and perinuclear cytoplasm of some ICs and SMCs, as well as in the urothelium (not shown).

Vimentin, CD34, and PDGFR*α* labeling of ICs were quantified in the bladder and urethra after both intermediate and chronic treatment with CYP, and they were compared to those in control animals (Figure 4). Intermediate CYP treatment induced a very pronounced increase in the intensity and extension of the immunoreactivity to the three markers used, evident in both the lamina propria and muscle layer of the bladder and urethra. After CYP chronic treatment, ir to these markers slightly increased relative to the controls or remained as it was after the intermediate treatment (Figure 4): vimentin/CD34 (Figures 1(b), 1(e), and 1(f)), vimentin/PDGFR*α* (Figures 2(b), 2(c), 2(e), and 2(f)–2(h)), and CD34/PDGFR*α* (Figures 3(b) and 3(d)–3(f)) double-labeling in both the bladder and urethra after intermediate and chronic CYP treatment.

The size of CD34^+^ cells significantly augmented after CYP-intermediate and CYP-chronic treatments in both the detrusor (from 75.4 ± 2.9 *μ*m^2^ to 208 ± 6.6 *μ*m^2^; *n* = 12, *P* < 0.0001; and 163 ± 3.4 *μ*m^2^; *n* = 12, *P* < 0.0001, resp.) and the urethra (from 67.6 ± 1.6 *μ*m^2^ to 161 ± 3.9 *μ*m^2^; *n* = 12, *P* < 0.0001; and 139 ± 2.3 *μ*m^2^; *n* = 12, *P* < 0.0001). Similar results were obtained with vimentin^+^ cells described in both the detrusor (from 85.7 ± 2.6 *μ*m^2^ to 219 ± 3.3 *μ*m^2^; *n* = 12, *P* < 0.0001; and 155 ± 2.2 *μ*m^2^; *n* = 12, *P* < 0.0001) and the urethra (from 91 ± 1.9 *μ*m^2^ to 190 ± 3.2 *μ*m^2^; *n* = 12, *P* < 0.0001; and 151 ± 3.3 *μ*m^2^; *n* = 12, *P* < 0.0001).

Both treatments provoked a significant increase in the colocalization index for each pair of markers (Table 1). Accordingly, these cells established broader cytoplasmic extensions, although these extensions had a somewhat fragmented appearance. This change was particularly evident with CD34 (Figures 1(b), 1(e), 1(f), 3(b), 3(e), and 3(f)) and vimentin (Figures 1(b), 1(f), 2(c), and 2(f)) staining.

Both intermediate and chronic CYP treatment increased the number and size of bipolar ICs parallel to muscle fibers (Figures 1(b), 2(b), 2(c), 2(h), and 3(b)) and those of the multipolar cells lying in between the muscle bundles (Figures 1(b), 2(h), and 3(b)). In addition, there was a higher density of large multipolar ICs in the lamina propria of both organs that accompanied the increase in thickness of this layer produced by the edema associated with cystitis (Figures 1(e), 1(f), 2(b), 2(c), 2(e), and 3(d)–3(f)). Into the serosa, at the periphery of the muscle bundles, the density of ICs increased, especially the vimentin- and CD34-immunoreactive ICs, albeit primarily in the urethra (Figure 1(f)).

Many of the larger cells labeled for vimentin but not for CD34 (Figures 1(b) and 1(f)) or PDGFR*α* (Figure 2(c)) were present in the serosal coat and lamina propria and in between the muscle fibers. The labeling in the urothelium and compact subepithelial cell layer (vimentin and PDGFR*α* in the bladder and only PDGFR*α* in the urethra) did not change with respect to the control conditions (Figures 1(a), 1(b), 2(a)–2(c), 3(a) and 3(b)).

### 3.2. Preventive Effect of Imatinib

Oral administration of Imatinib (10 mg kg^−1^) five days before and during CYP treatment significantly dampened the effect of CYP on the IC populations in both the lamina propria and smooth muscle layer of the bladder and urethra (Figure 4). The decrease in IC number was accompanied by a significant reduction in the colocalization index, also suggesting a reduction in the extension of the labeling within the cells (Table 1). This reduced cell density was evident after intermediate CYP treatment (Figures 3(c), and 4) but it was more dramatic after the chronic one, leading to the practical disappearance of IC labeling for the three markers used (Figures 1(d) and 4). In striking contrast to ICs, CD34-immunoreactive cells in the urothelium and vimentin- and PDGFR*α*-immunoreactive cells in the subepithelial coat were unaffected by Imatinib treatment (Figures 1(c), 1(d), and 3(c)). Moreover, the density of ICs did not change significantly in the control animals that received Imatinib alone (Figure 1(c)).

**Figure 5:**
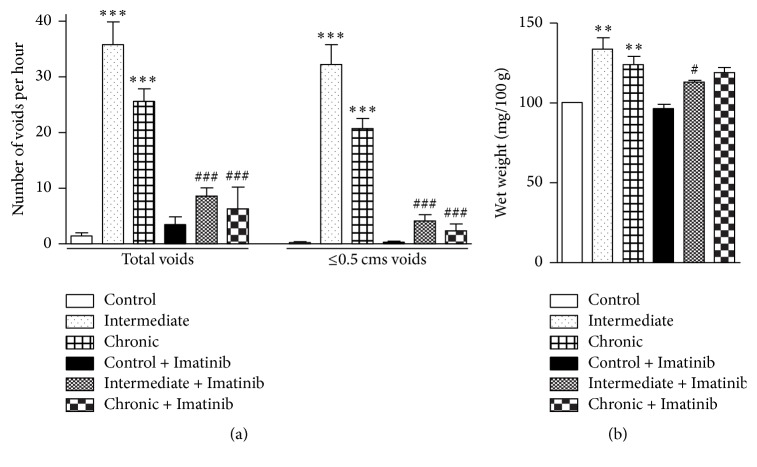
Changes in voiding frequency and in urinary tract wet weight in CYP-treated rats and the reversion following Imatinib administration. The total number and the number of small-volume voids per hour were measured (a), as well as the complete lower urinary tract wet weight after sacrificing the animals (b). Data are expressed as the mean ± SEM (*n* = 6-7 per group): ^*∗∗*^*P* < 0.01 and ^*∗∗∗*^*P* < 0.001 compared to controls; ^#^*P* < 0.05 and ^###^*P* < 0.001 compared with the corresponding treatment without Imatinib (one-way ANOVA followed by Dunnett's multiple comparison test).

As previously described [4], CYP treatment significantly altered the micturition frequency by producing a substantial increase in the total number of voids and in particular in the number of small-volume voids (Figure 5(a)). These changes were significantly impeded by pretreatment with Imatinib, which also minimized the increase in lower urinary tract wet weight that accompanied intermediate CYP treatment (Figure 5(b)).

### 3.3. Induction of eNOS Expression in ICs by CYP and Its Reversion by Imatinib

**Figure 6:**
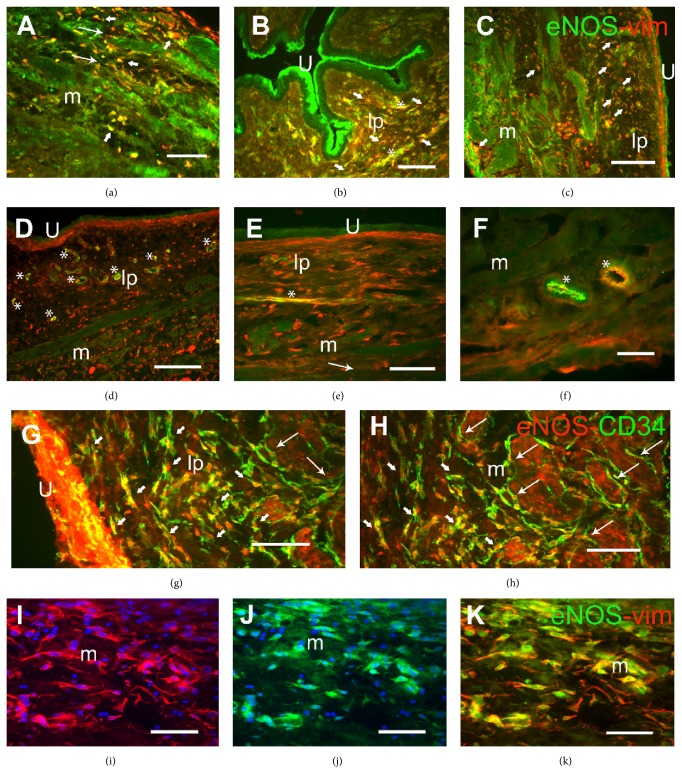
The eNOS expression induced by CYP in ICs within the rat bladder and urethra and the preventive effect of Imatinib. (a–f) Representative images showing the labeling in the urethra for eNOS (green) and vimentin (vim, red) in the muscle layer after CYP-intermediate (a) and in the lamina propria after CYP-chronic treatment (b). Similar labeling in the bladder of CYP-chronic (c), control (d), Imatinib-intermediate (e), and Imatinib-chronic (f) rats. The colocalization of eNOS with vimentin was only evident in CYP-treated animals (a–c), while it was restricted to the endothelium in controls (d) and in animals pretreated with Imatinib (e, f). (g-h) Representative images showing eNOS (red) and CD34 (green) colabeling of ICs in the lamina propria (g) and the muscle layer (h) of CYP-intermediate treated bladders. (i–k) Labeling for vimentin (red, (i)) and eNOS (j) and the merged image (k) of ICs in the lamina propria of a CYP-intermediate treated bladder. The nuclei were counterstained with DAPI (blue) in (i) and (j). Long arrows indicate double-labeled bipolar cells with long prolongations. Short arrows show double-labeled multipolar cells located between muscle fibers and in the lamina propria. Asterisks indicate the labeling of intramural vessels. U, urothelium; lp, lamina propria; m, muscle layer. Scale bars = 40 *μ*m except in (c) and (d) (20 *μ*m) and (f) (80 *μ*m).

In ICs with an elongated morphology, scant cytoplasm, and long cell processes, eNOS expression only colocalized with vimentin (Figures 6(a)–6(c)) and CD34 (Figures 6(g)–6(k)) after CYP treatment, both in the bladder (Figures 6(c), 6(g), and 6(h)) and in urethra (Figures 6(a) and 6(b)). However, in control animals eNOS antibodies did not recognize vimentin- or CD34-immunoreactive ICs, and eNOS was only detected in the endothelium of the intramural vessels (asterisks in Figure 6(d)). Pretreatment with Imatinib prevented eNOS expression in the CD34- and vimentin-immunoreactive ICs within the bladder of rats treated with CYP for 48 h, and eNOS-ir was again restricted to the endothelium (asterisk in Figures 6(e) and 6(f)). The lack of suitable antibodies generated in a species other than rabbit, in which the eNOS antibody was raised, impeded the direct comparison between eNOS and the other ICs marker, PDGFR*α*.

## 4. Discussion

The present study shows that intermediate and chronic treatment with CYP produce a significant increase in the density of ICs within the rat bladder and urethral wall, cells labeled by the following pairs of markers: vimentin/CD34; vimentin/PDGFR*α*; and CD34/PDGFR*α*. Additionally, IC proliferation was accompanied by hypertrophy, an increase in cell size that could explain the higher colocalization index observed for the three pairs of markers seen in the CYP-treated rat bladder and urethra. Hyperplasia of CD34-immunoreactive ICs has been previously described being associated with inflammation and tumoral processes and in myxoid and edematous lesions [21–23]. In many of these cases, IC proliferation is accompanied by acquisition of a voluminous nucleus and cytoplasm, with wider and shorter prolongations [22, 24]. Together these changes suggest IC activation by inflammation.

While c-kit-ir is considered the gold standard to identify ICCs in the gastrointestinal tract, it is now accepted that c-kit-ir is not a specific feature of ICs in other hollow organs [25]. Indeed available c-kit-antibodies fail to identify ICs in frozen bladder and urethra sections from different species, including rat, mouse, and humans [8, 26–30]. Dual CD34/PDGFR*α* immunolabeling and a lack of c-kit-ir are considered specific for a second population of ICs within the gastrointestinal tract, where ICCs are c-kit^+^ and CD34^−^/PDGFR*α*^−^ [25]. CD34^+^/c-kit^−^ cells were also described in the human bladder and referred to as interstitial Cajal-like cells (ICLCs) [31], cells probably corresponding to the so-called fibroblast-like cells in the mouse bladder (PDGFR*α*^+^) [27]. Other studies used the term telocytes (TCs) to refer to CD34^+^/PDGFR*α*+ cells in the lamina propria of the human pelvis, ureter, urethra [26], and bladder [30] with specific ultrastructural features such as long cytoplasmic prolongations named telopodes. There is still debate about the most appropriate term to denominate these ICs in the urinary tract.

This confusing nomenclature explains why not all the cells referred to as “fibroblasts” or “fibroblast-like cells” are real fibroblasts but, rather, they could potentially be ICs [32]. However, it is accepted that CD34/vimentin and CD34/PDGFR*α* ir are distinctive features of ICs that are not shared by fibroblasts, which could be vimentin^+^ and PDGFR*α*^+^ but not CD34^+^ [33]. Thus, our results support the notion that cells which proliferate in response to CYP-induced cystitis in the rat bladder and urethra are mainly ICs. Beside this predominant IC type, present in the lamina propria, as well as in between muscle fibers, another cell population of ICs densely packed just beneath the bladder and urethral urothelium. These cells were PDGFR*α*^+^ and vimentin^+^ but CD34^−^. The role this compact subepithelial layer has received considerable attention lately, having been proposed to modulate afferent discharge and sensations [10, 11]. Whether these cells are ICs with a different phenotype [30] or fibroblasts with myoid differentiation, given the expression of *α*-smooth muscle actin [34], awaits further investigations. In the present study, these suburothelial ICs in the bladder and urethra were not affected by either CYP or Imatinib treatment. Hence, these ICs appear to be different to the prevalent ICs in the bladder and urethral wall.

In inflammation, granulation tissue forms to repair the lesion. These changes occur when CYP induces cystitis in the rat, which provokes edema, hemorrhage, and progressive infiltration of CD163^+^ macrophages, and it augments with the duration of the treatment [4]. Stromal cells, both resident and hematopoietic-derived, are considered to be active in inducing and maintaining inflammatory process [35]. In many instances, the arrival of stromal cells, especially fibroblasts, gives rise to myofibroblasts that are involved in interstitial fibrosis [36]. It has even been suggested that intercellular signaling may change the phenotype of ICs, transforming them into matrix secreting myofibroblasts, as supported by the loss of CD34 expression and the gain of *α*-smooth muscle actin (a distinctive feature of myofibroblasts [37]). In bladder pain syndrome, IC distribution is altered in conjunction with a phenotypic transformation to more fibroblast-like cells [38]. No phenotypic change was observed here as CYP-induced cystitis progressed, and the prominent increase in CD34-ir during the intermediate and chronic inflammatory process further suggests that proliferating cells cannot be considered myofibroblasts.

A robust correlation between urinary bladder dysfunction and ICs has been previously documented. An increase in ICs was reported in a model of partial bladder outlet obstruction that led to OAB [12] and in the overactive human bladder [15]. Moreover, an altered distribution or phenotypic disturbances of ICs may contribute to the pathophysiology of OAB [18]. Upregulation of connexin-43 associated with ICs in human and rat neurogenic bladders is consistent with changes in signal transmission through the IC network in pathological conditions, possibly enhancing bladder excitability [39]. In CYP-treated rats, denser ICs were located at the periphery of muscle bundles and in the lamina propria, forming a network that could allow ICs to coordinate signals between the urothelium and intramural nerves to the muscle and vice versa. Thus, a higher density of ICs could augment bladder excitability and in turn trigger OAB.

Overproduction of NO or NO synthase-derived free radicals has long been known to fulfill pivotal roles in CYP-induced cystitis [1, 4]. Here we demonstrate de novo expression of eNOS in vimentin- and CD34-immunoreactive cells in the bladder and urethral wall of CYP-treated animals. This further supports our previous data indicating that eNOS expression, but not iNOS, is upregulated in the rat bladder and urethra following CYP treatment [4]. Therefore, under CYP treatments such expression of eNOS in a boosted IC population, together with its concomitant overexpression in smooth muscle, could lead to NO overproduction, thereby contributing to the inflammatory process.

It is noteworthy that pretreatment with Imatinib significantly inhibited the proliferation of ICs in the bladder and urethra, and it prevented the pronounced increase in voiding frequency and in the wet weight of the urinary tract following CYP administration. Furthermore, Imatinib impairs de novo eNOS expression by ICs in CYP-treated animals. Hence, IC proliferation, eNOS expression, and bladder hyperactivity would all appear to be interrelated processes. In the lower urinary tract, Imatinib reduces both spontaneous and inducible bladder activity and it improves bladder compliance, capacity, and voiding frequency during guinea-pig cystometry [15, 17, 40]. It was postulated that Imatinib acts by directly inhibiting the function of ICs, blocking the transmission of cholinergic signals from the autonomic nerves to the detrusor muscle [19]. Remarkably, inhibitory effects of Imatinib on bladder contractility seem to be more intense on the overactive human detrusor [15]. Indeed, it was recently shown that Imatinib significantly reduces the number of ICs in rat neonatal bladders in conjunction with reduced muscarinic contractions in isolated strips from these bladders [34]. These effects of Imatinib suggest that it may have possible therapeutic applications in the prevention of the bladder disorders that accompany chemotherapy with CYP.

While the effect of CYP treatment on the bladder is well documented, little is known about its effect on the urethra and the contribution of this organ to the overall OAB symptomatology. Now it is clear that CYP treatment induces similar inflammatory conditions in the bladder as in the urethra [4]. Furthermore, ICs proliferates and Imatinib prevents these effects in a similar way in both organs (present work). Therefore, we should consider that CYP treatment induces urethritis together with cystitis and that both processes contribute to the pathological condition.

## 5. Conclusions

In conclusion, we show that the inflammatory reaction induced by CYP in the bladder and urethra is accompanied by a prominent increase in the density of ICs that express vimentin, CD34, and PDGFR*α*. These changes occur in conjunction with the appearance of marked bladder hyperactivity and these cells only express eNOS under CYP-induced inflammatory conditions. ICs may represent a new therapeutic target to reduce inflammatory conditions of the urinary tract. Indeed, Imatinib effectively inhibits all these changes, suggesting possible therapeutic benefits in preventing the secondary effects in the bladder during chemotherapy with CYP. Furthermore, Imatinib could potentially be used as a specific inhibitor of the biological function of ICs in animal models or in primary cultures of ICs from different tissues. A deeper understanding of how ICs communicate with neighboring cells and influence signaling pathways during inflammation will be essential to identify novel therapeutic strategies.

## Figures and Tables

**Table 1:** Colocalization of the different IC markers in the lamina propria and muscle layer of the rat bladder and urethra, under control conditions, following intermediate and chronic CYP treatment, and after prior Imatinib treatment.

	Control		Intermediate		Intermediate + Imatinib		Chronic		Chronic + Imatinib	
	Lamina propria	Muscle layer	Lamina propria	Muscle layer	Lamina propria	Muscle layer	Lamina propria	Muscle layer	Lamina propria	Muscle Layer
*Bladder*										
VIM/CD34	0.67 ± 0.01	0.71 ± 0.01	0.75 ± 0.01^*∗∗∗*^	0.75 ± 0.01^*∗∗*^	0.69 ± 0.01^##^	0.68 ± 0.02^###^	0.79 ± 0.01^*∗∗∗*^	0.80 ± 0.01^*∗∗∗*^	0.62 ± 0.01^*∗∗∗*,###^	0.61 ± 0.01^*∗∗∗*,###^
VIM/PDGFR*α*	0.72 ± 0.01	0.71 ± 0.01	0.79 ± 0.01^*∗∗∗*^	0.77 ± 0.02^*∗∗∗*^	0.70 ± 0.01^###^	0.69 ± 0.01^*∗*###^	0.80 ± 0.01^*∗∗∗*^	0.81 ± 0.01^*∗∗∗*^	0.63 ± 0.01^*∗∗∗*,###^	0.62 ± 0.01^*∗∗∗*,###^
CD34/PDGFR*α*	0.80 ± 0.01	0.81 ± 0.01	0.85 ± 0.01^**∗∗∗**^	0.85 ± 0.01^*∗∗∗*^	0.83 ± 0.01^*∗∗*,##^	0.80 ± 0.01^###^	0.83 ± 0.01^*∗∗*^	0.84 ± 0.01^*∗*^	0.75 ± 0.01^*∗∗*,###^	0.75 ± 0.01^*∗∗*,###^

*Urethra*										
VIM/CD34	0.67 ± 0.01	0.69 ± 0.01	0.74 ± 0.01^*∗∗∗*^	0.73 ± 0.01^*∗∗*^	0.69 ± 0.01^###^	0.67 ± 0.01^*∗*,###^	0.76 ± 0.01^*∗∗∗*^	0.78 ± 0.01^*∗∗∗*^	0.61 ± 0.01^*∗∗∗*,###^	0.60 ± 0.01^*∗∗∗*,###^
VIM/PDGFR*α*	0.70 ± 0.01	0.72 ± 0.01	0.75 ± 0.01^*∗∗∗*^	0.76 ± 0.01^*∗∗∗*^	0.67 ± 0.01^###^	0.67 ± 0.01^ ###^	0.78 ± 0.01^*∗∗∗*^	0.79 ± 0.01^*∗∗∗*^	0.65 ± 0.01^*∗∗∗*,###^	0.65 ± 0.01^*∗∗∗*,###^
CD34/PDGFR*α*	0.69 ± 0.01	0.69 ± 0.01	0.78 ± 0.01^*∗∗∗*^	0.77 ± 0.01^*∗∗∗*^	0.69 ± 0.01^###^	0.70 ± 0.01^###^	0.75 ± 0.01^*∗∗*^	0.77 ± 0.01^*∗∗∗*^	0.67 ± 0.01^*∗*,###^	0.66 ± 0.01^*∗*,###^

Animals were treated with CYP for 48 h (intermediate: 150 mg kg^−1^) and 10 days (chronic: 50 mg kg^−1^ every third day) and compared with controls and after pretreatment with Imatinib five days before and during CYP treatments (10 mg kg^−1^, oral administration). The different antibodies were assayed in pairs by dual immunofluorescence and colocalization results are expressed as a correlation index (ranged from 0 to 1) which indicates the positively correlated pixels between both antibodies. Values represent the mean ± SEM (*n* = 6–8 different fields from at least 4 different animals). ^*∗*^*P* < 0.05, ^*∗∗*^*P* < 0.01, and ^*∗∗∗*^*P* < 0.001 compared to controls; ^##^*P* < 0.01 and ^###^*P* < 0.001 compared with the corresponding treatment without Imatinib (one-way ANOVA followed by Dunnett's multiple comparison test).
